# Lithium Controls Central Nervous System Autoimmunity through Modulation of IFN-γ Signaling

**DOI:** 10.1371/journal.pone.0052658

**Published:** 2012-12-28

**Authors:** Amber L. Rowse, Rodrigo Naves, Kevin S. Cashman, Donald J. McGuire, Tethia Mbana, Chander Raman, Patrizia De Sarno

**Affiliations:** 1 Department of Psychiatry and Behavioral Neurobiology, University of Alabama at Birmingham, Birmingham, Alabama, United States of America; 2 Department of Microbiology, University of Alabama at Birmingham, Birmingham, Alabama, United States of America; 3 Department of Medicine, University of Alabama at Birmingham, Birmingham, Alabama, United States of America; Escola Paulista de Medicina - UNIFESP, Brazil

## Abstract

Inhibitors of glycogen synthase kinase 3 (GSK3) are being explored as therapy for chronic inflammatory diseases. We previously demonstrated that the GSK inhibitor lithium is beneficial in experimental autoimmune encephalomyelitis (EAE), the mouse model of multiple sclerosis. In this study we report that lithium suppresses EAE induced by encephalitogenic interferon-γ (IFN-γ)-producing T helper (Th1) cells but not by interleukin (IL)-17-producing T helper (Th17) cells. The therapeutic activity of lithium required functional IFN-γ-signaling, but not the receptor for type I IFN (IFNAR). Inhibitor/s of GSK3 attenuated IFN-γ dependent activation of the transcription factor STAT1 in naïve T cells as well as in encephalitogenic T cells and Th1 cells. The inhibition of STAT1 activation was associated with reduced IFN-γ production and decreased expansion of encephalitogenic Th1 cells. Furthermore, lithium treatment induced *Il27* expression within the spinal cords of mice with EAE. In contrast, such treatment of *Ifngr^−/−^* mice did not induce *Il27* and was associated with lack of therapeutic response. Our study reveals a novel mechanism for the efficacy of GSK3 targeting in EAE, through the IFN-γ-STAT1 axis that is independent IFNAR-STAT1 axis. Overall our findings set the framework for the use of GSK3 inhibitors as therapeutic agents in autoimmune neuroinflammation.

## Introduction

Multiple sclerosis (MS) is an autoimmune neurodegenerative disease in which both adaptive and innate immunity play a role. CD4^+^ T cells, believed to be early effector cells in the disease, migrate to the central nervous system (CNS), leading to demyelination, axonal loss, and neurological disability. The cells of the innate immune system are also involved both in the initiation and progression of MS by influencing the effector function of T cells [1], [2]. Both Th1 and Th17 cells are involved in the pathogenesis of MS, and are the primary effector cells in experimental autoimmune encephalomyelitis (EAE), the most common animal model of MS [3]–[6]. These lineages have distinct effector functions and are characterized by the expression of specific transcription factors and cytokines. The differentiation of naïve CD4^+^ T cells to interferon-γ (IFN-γ)-producing T helper (Th1) cells is dependent on IFN-γ and interleukin (IL)-12, activation of STAT1 and STAT4, respectively, and the transcription factor Tbet [7]. TGF-β and IL-6, and STAT3 drive IL-17-producing T helper (Th17) cell differentiation in a process that is dependent on the transcription factor ROR-γt [8], [9]. Although IL-23 is not needed for differentiation, it has an essential role in pathogenicity of Th17 cells perhaps by promoting expansion and stability [10].

The IFN-γ-STAT1 signaling axis has an important pleiotropic role, both pathogenic and protective, in autoimmune diseases including MS and its mouse model, EAE [11]. Both Th1 and Th17 cells are independently capable of inducing autommunity in mouse models and they not only play a role in regulating one another, but that they have a more complex, both overlapping and differential, role in tissue inflammation [4], [12], [13]. There is also increasing evidence of the plasticity/instability of the Th17 cell phenotype; Th17 cells may acquire Tbet expression, gaining the ability to secrete IFN-γ in addition to IL-17 [14]. These dual cytokine expressing Th17 cells may ultimately lose the ability to secrete IL-17 and convert into Th1-like cells. Thus the finding that Th17 cells can turn into Th1 cells highlights the importance of controlling the effector function of Th1 cells once disease is established.

We have recently found that relapsing-remitting MS segregates into a Th1 or a Th17 disease and that each form of disease is differentially responsive to type I IFN therapy [15]. Thus the elucidation of signaling pathways regulating the production and expansion of specific Th effector cells in EAE and MS is a necessary goal to identify new specific targets for therapeutic intervention. A lot is known about the transcription factors and cytokines that are determinant for the differentiation of Th1 and Th17 effector cells, but the mechanisms regulating their production, expansion and pathogenic function in disease are still largely undefined.

GSK3 is a constitutively active serine/threonine kinase that is a critical modulator of innate and adaptive immunity through the regulation of several transcription factors important in the production of cytokines and inflammation, including NF-kB, CREB, AP-1 and STATs [16]. We have previously shown that the GSK3 inhibitor lithium is prophylactic and therapeutic in EAE [17]. Recovery from EAE in lithium treated mice was associated with reduced demyelination, reduced microglia activation, and reduced CD4^+^ T cell infiltration in the spinal cord. We also found that treatment of mice *in vivo* with the GSK3 inhibitor lithium, inhibited myelin oligodendrocyte glycoprotein peptide (MOG_35–55_)-specific T cell proliferation and significantly reduced MOG_35–55_-specific production of IFN-γ, IL-6, and IL-17 from splenocytes [17]. GSK3 has been shown to facilitate IFN-γ mediated activation of macrophages [18]. Furthermore inhibition of GSK3 in macrophages suppresses activation of STAT3 and STAT5, and constrains the synergistic activation by IFN-γ and lipopolysaccharides (LPS) of STAT3 [19], [20]. However the mechanism of the therapeutic action of lithium in neuroinflammation *in vivo* is still unresolved. In this study we tested the hypothesis that lithium is beneficial in EAE through GSK3 regulation of IFN-γ signaling. Our results show that lithium suppresses Th1 but not Th17 neuroinflammation, and through inhibition of GSK3 tunes IFN-γ-STAT1 signaling for optimal therapeutic efficacy in EAE.

## Materials and Methods

### Ethic Statement

All experimental animal work in this study was conducted in strict accordance with the National Institutes of Health and University of Alabama at Birmingham Institutional Animal Care and Use Committee (IACUC) guidelines. The protocol was approved by the IACUC of the University of Alabama at Birmingham (approval number 111208672). All Surgery was performed under isofluorane anesthesia, and all efforts were made to minimize suffering.

### Mice

C57BL/6 mice were purchased from Frederick Cancer Research. B6.129S7-*Ifngr1* ^tm/Agt^/J (*Ifngr1^−/−^*) mice were purchased from the Jackson Laboratory and backcrossed onto C57BL/6 background for 10–12 generations. C57BL/6 *Stat1^−/−^*, *Ifnar1^−/−^*, and IL-17F-Thy1.1 reporter mice [14] were kind gifts from R. Lorenz, J.D. Mountz, and C. Weaver, respectively (UAB). For lithium treatment, lithium was administered in pelleted food containing 0.2% lithium carbonate (Harlan-Teklad) as previously described [17]. This lithium administration is used to achieve serum levels equivalent to those attained therapeutically in human patients.

### In vitro Stimulations

Macrophages were lavaged from the peritoneum on day 4 after injection with Brewer’s thioglycollate. Mononuclear cells were isolated from spleen using the standard protocol of first mashing the spleen through a cell strainer, then lysing red blood cells by using ACK (Ammonium-Chloride-Potassium) Lysing Buffer, and then washing well the cells with PBS, and re-suspending them in culture medium. Mononuclear cells were stimulated with 5 U/ml IFN-γ or 100 U/ml IFN-β (Biolegend) and/or 1.25 µg/ml anti-CD3 (145-2C11). Where indicated, cultures were supplemented with GSK3 inhibitors LiCl (5 mM-20 mM; Sigma) or TDZD-8 (5 µM; Calbiochem).

### Flow Cytometry

Cells were stained with anti-CD4 (RM4-5; Biolegend), fixed, permeabilized with Phosflow Perm Buffer III (BD Pharmingen) and stained with an antibody against phospho-STAT1-Y701 (p-STAT1) (58D6, Cell Signaling). For intracellular cytokine staining, cells were incubated with Brefeldin A, 1X as recommended by manufacturer (Biolegend), 50 ng/ml PMA (Sigma-Aldrich) and 500 ng/ml ionomycin (Sigma-Aldrich) for 4 h. Cells were stained with anti-CD4 (RM4-5; eBioscience), fixed, permeabilized (Biolegend) and then stained with antibodies against IFN-γ (XMG1.2; eBioscience) or IL-17A (TC11-18H10.1; Biolegend). Data was collected on an LSRII (BD) and analyzed using FlowJo (TreeStar).

### Induction of Active EAE

For active EAE, male mice were immunized with a s.c. injection of 50 µg MOG_35–55_ emulsified in incomplete Freund’s adjuvant (Difco) containing 125 µg *M. tuberculosis* (H37Ra; Difco). Immunized mice were monitored for classical disease using a standard scale of 0 to 6∶0, no clinical signs; 1, loss of tail tone; 2, flaccid tail; 3, incomplete paralysis of one or two hind legs; 4, complete hind limb paralysis; 5, moribund (animals were humanly euthanized); 6, death. Atypical EAE in *Ifngr1^−/−^* mice was scored on a 0–6 scale: 0, no disease; 1, slight head tilt; 2; severe head tilt, 3; slight axial rotation/staggered walking, 4; severe axial rotation/spinning; 5, moribund; 6, death. Scores reported for *Ifngr1^−/−^* mice are classical and atypical combined.

### *Ex-vivo* Encephalitogenic T Cell Restimulation, Polarizations and Adoptive Transfer of EAE

Mononuclear cells from spleens and draining lymph nodes (dLNs) of MOG_35–55_-immunized WT mice were restimulated with 10 µg/ml MOG_35–55_ (CPC Scientific) for 24 h. For the generation of encephalitogenic Th1 cells, cells were cultured with 10 µg/ml MOG_35–55_, 20 ng/ml IL-12 (Biolegend) and 1 µg/ml anti-IL-4 neutralizing antibody for 3 days. On day 2, 2.5 ng/ml IL-2 (Biolegend) was added to the culture. Where indicated, Th1 cells were restimulated with anti-CD3 and anti-CD28 (1 µg/ml, each) for 8 h and supernatants were assayed for IFN-γ production by ELISA (Biolegend). For the generation of encephalitogenic Th17 cells, cells were cultured with 20 ng/ml IL-23 (Biolegend), 10 µg/ml anti-IFN-γ and 1 µg/ml anti-IL-4 neutralizing antibodies for 3 days. Where indicated, polarized Th17 cells were cultured without or with LiCl for additional 24 h and supernatants were assayed for IL-17A and GM-CSF production by ELISA (eBioscience).

For adoptive transfer of EAE, donor mice were immunized with a s.c. injection of 150 µg MOG_35–55_ emulsified in complete Freund’s adjuvant. Mononuclear cells from spleens and dLNs of MOG_35–55_-immunized mice were restimulated with 10 µg/ml MOG_35–55_ under either Th1 or Th17 polarizing conditions (described above). Lithium-treated recipient mice were fed lithium chow 6–10 days prior to transfer. Cells (4–6×10^6^) were injected i.v. into 350 rad irradiated untreated or lithium-treated recipient mice. Cells secreting IL-17F were isolated from IL-17F-Thy1.1 mice, cultured under Th17 polarization conditions (as above), labeled with biotin anti-rat CD90/MUCD90.1 (OX-7; Biolegend) and magnetically sorted using Dynabeads biotin binder (Invitrogen Dynal). Enriched cells (3–6×10^5^) were injected i.v. as above. Mononuclear cells were isolated, following perfusion with PBS, from the spinal cords of untreated and lithium-treated Th1 animals. Spinal cords were incubated with 2 mg/ml collagenase D (Roche) and 5 U/ml DNase (Sigma-Aldrich) for 1 h at 37°C. Mononuclear cells from the spinal cord were purified by two-step Percoll gradient centrifugation as done previously [15], [17], and described in detail in [21].

### RNA Isolation and RT-PCR

Spinal cords were isolated from EAE mice perfused with PBS on day 20 post-immunization and snap frozen using dry ice in ethanol. RNA was extracted using Trizol reagent (Invitrogen) and cleaned up using RNeasy Mini Kit (Qiagen). cDNA synthesis was performed using SuperScript VILO cDNA synthesis kit (Invitrogen) per manufacturer’s instruction. Gene expression was assayed using Taqman Gene Expression Assays (Applied Biosystems) in combination with Taqman Fast Advanced Master Mix (Applied Biosystems). Taqman assay IDs include: *Hprt* (Mm00446968_m1), *Il10* (Mm00439614_m1), *Il27* (Mm00461164_m1), *Nos2* (Mm00440502_m1) and *Ifnb1* (Mm00439552_s1). Expression data are normalized to *Hprt* and expressed as 2^−Δ*C*^ *_T_*; [2^−*C*^ *_T gene of interest_* ^−*C*^ *_T hprt_*].

### Statistics

Results were analyzed by *t*-test or one-way ANOVA, as appropriate. For analysis of the EAE clinical scores curves and day of onset, the non-parametric Mann-Whitney test was used.

## Results and Discussion

### Lithium Attenuates Th1 EAE and not Th17 EAE

**Figure 1:**
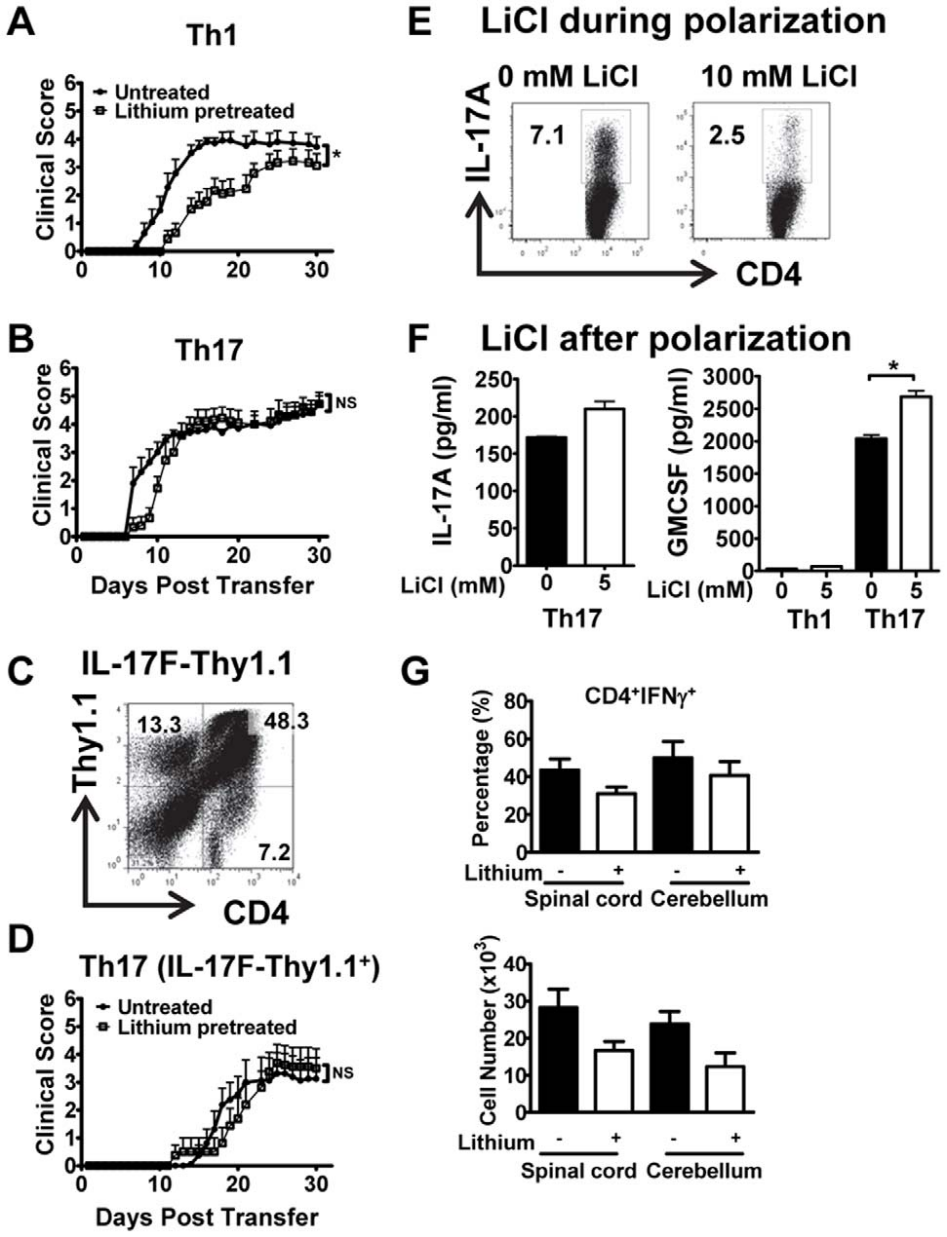
Lithium inhibits Th1-induced, but not Th17-induced EAE. (A) Th1 cells (4–6×10^6^), (B) Th17 cells (4–6×10^6^), or (C and D) IL-17F-Thy1.1 cells (3–6×10^5^) were adoptively transferred i.v. into naïve untreated or lithium–treated recipient mice to induce EAE (mean ± SEM, *n* = 8–11 mice/group, *p<0.05 or NS, not significant, as determined by Mann-Whitney). (E) Intracellular cytokine staining for IL-17A in CD4^+^ gated cells from MOG_35–55_-immunized mice polarized to Th17 for 3 days in the absence or presence of 10 mM LiCl (representative dot plots and percentages are shown, *n* = 2). (F) 3 days polarized Th17 or Th1 cells from MOG_35–55_-immunized mice were cultured for an additional 24 h without or with addition of 5 mM LiCl, and production of IL-17A and GM-CSF was measured by ELISA in culture supernatants (one representative experiment is shown, *n* = 2; *p<0.05). (G) Infiltration of Th1 cells (CD4^+^IFN-γ^+^) into the spinal cord and cerebellum of untreated or lithium pretreated mice with adoptive transferred EAE. Infiltrating cells were isolated (14–15 d post transfer) and characterized by flow cytometry. Bar graphs depict percentage (top) and absolute number (bottom) of CD4^+^IFN-γ^+^ cells. Results are from 2–3 mice pooled per experiment (*n* = 2).

IFN-β, the major therapy for the treatment of MS, inhibits the differentiation of naïve T cells to Th17 cells [22]–[25]. However, we have found that it is ineffective in treating MS patients with a Th17 signature or mice with a Th17 form of EAE [15]. Thus in the present study we interrogated if lithium can equally attenuate both Th1 and Th17 disease, an important question since lithium is therapeutic in EAE [17] and inhibits *in vitro* differentiation of naïve T cells to Th17 cells [26]. We observed that in lithium-treated animals EAE induced by encephalitogenic Th1 cells was significantly delayed in onset (Table 1) and less severe than that in untreated mice (Fig. 1A). In contrast, Th17 EAE in lithium treated mice, although slightly delayed in onset (Table 1), had the same disease severity as in untreated mice (Fig. 1B). We confirmed this result, using highly enriched encephalitogenic Th17 from immunized IL-17F-Thy1.1 reporter mice that were sorted for Thy1.1 expression after Th17 polarization (Figs. 1C, 1D) [14]. This approach enabled us to exclude contaminating Th1 cells as well as normalize for absolute numbers of IL-17-producing cells with that of IFN-γ-producing cells used for Th1 transfer. The lack of therapeutic benefit in Th17-induced EAE was surprising, considering the observation that lithium inhibits differentiation of naïve T cells to Th17 cells [26]. We suggest that this reflects a differential effect of lithium in polarized Th17 cells versus Th17 cells during differentiation and/or expansion. In fact, we did observe that lithium attenuated the expansion of Th17 cells when *in vivo* primed T cells were cultured with MOG_35–55_ under Th17 polarizing conditions (Fig. 1E). Furthermore, lithium treatment of Th17 cells, initiated after cells were already polarized for 3 days, resulted in increased GM-CSF production and no decrease in IL-17A secretion compared to untreated controls (Fig. 1F). GM-CSF is a key pathogenic determinant of encephalitogenic Th17 cells and its elevation by lithium treatment is consistent with treatment inefficacy [27], [28]. The inhibition of GSK3 by lithium may also promote pathogenicity of Th17 cells by increasing responsiveness to IL-17A and IL-17F and interrupting the GSK3-dependent feedback regulation of IL-17R signaling [29].

**Table 1:** Analysis of disease parameters for adoptive transfer of EAE induced by Th1, Th17, and IL-17F-Thy1.1 cells in untreated and lithium-treated animals.

	LiCO_3_diet	Incidence (%)	Onset (d)	Accumulative score
**Th1**	–	100% (11/11)	11.6±0.7	58.1±5.2
	+	100% (9/9)	16.2±1.4^a^	33.1±3.3
**Th17**	–	100% (10/10)	9.1±0.5	81.4±2.9
	+	100% (9/9)	11.4±0.6^b^	75.9±7.8
**IL-17F-Thy1.1**	–	88% (7/8)	19.4±1.2	37.9±9.4
	+	88% (7/8)	20.5±1.4	36.7±9.5

Data are presented as mean ± SEM (*n* = 8–11 mice).

a *p*<0.05; Lithium-treated Th1 compared to untreated Th1.

b *p*<0.05; Lithium-treated Th17 compared to untreated Th17.

The attenuation of Th1-induced EAE by lithium was associated with fewer CD4^+^IFN-γ^+^ cells infiltrating the spinal cord, in proportion and absolute number, compared to untreated Th1 mice (Figs. 1 G, Fig. S1). Although the number of Th1 cells was decreased, the absolute numbers of all infiltrating cells in the spinal cord or brain were slightly increased or unaltered (Fig. S1). However these differences were not statistically significant.

### Lithium Attenuates IFN-γ-induced STAT1-Y701 Phosphorylation in CD4^+^ T Cells through Inhibition of GSK3

**Figure 2:**
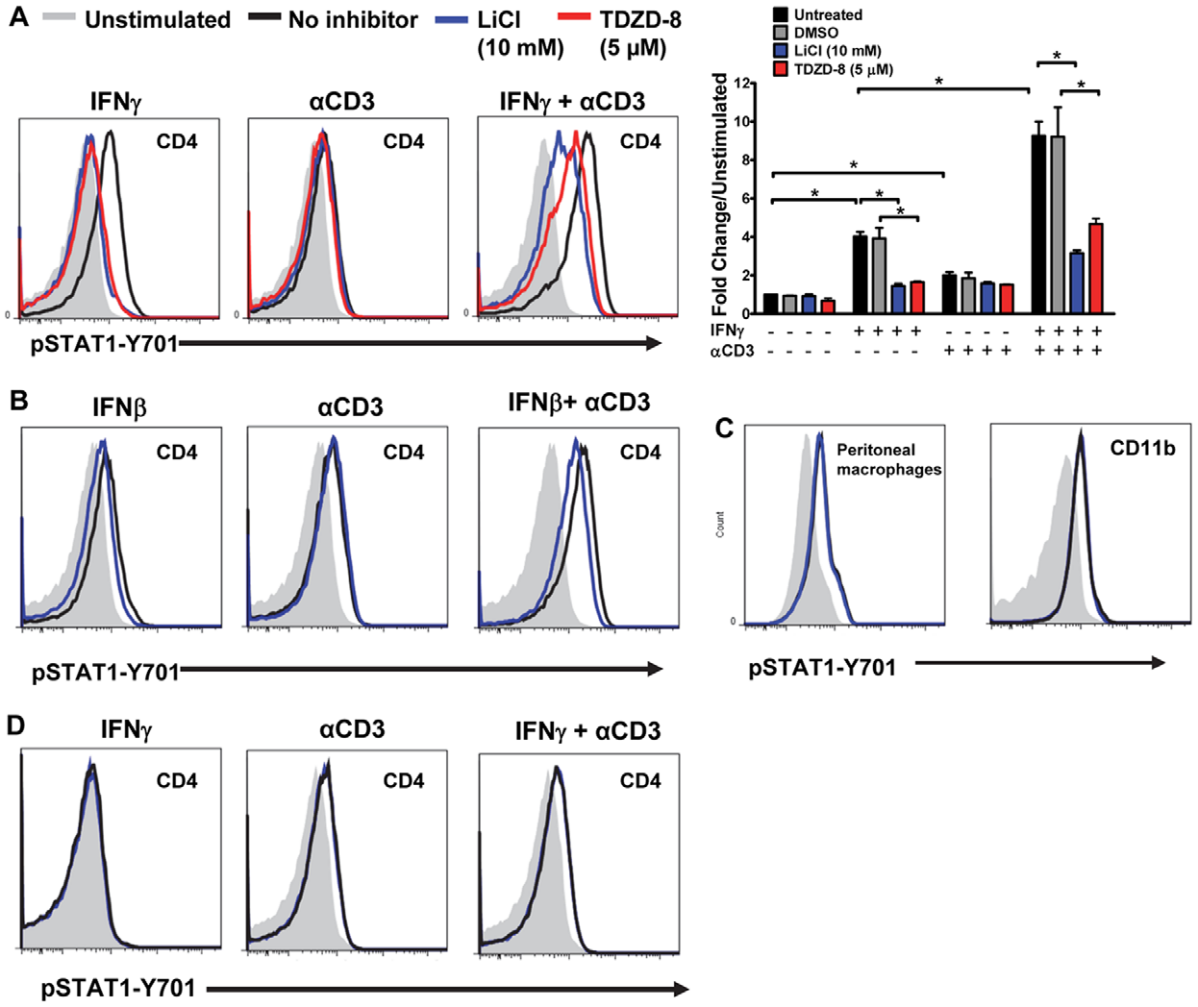
GSK3 promotes IFN-γ and IFN-β-induced STAT1-Y701 phosphorylation in CD4^+^ T cells. (A and B) Splenocytes were pre-incubated for 1 h in the absence or presence of the GSK3 inhibitors LiCl or TDZD-8, and stimulated without or with IFN-γ (5 U/ml; 25 minutes) or IFN-β (100 U/ml; 45 minutes) and/or anti-CD3 (1.25 µg/ml; 25 minutes in (A), and 45 minutes in (B) as indicated. Mononuclear cells were stained for pSTAT1-Y701 and analyzed by flow cytometry. Representative histograms are gated on CD4^+^ T cells. Induction of pSTAT1-Y701 is normalized to unstimulated cells. Fold induction of pSTAT1-Y701 MFI is normalized to unstimulated cells from combined data of 2-4 experiments. *p<0.05, as determined by one-way ANOVA. (C) Thioglycollate-elicited macrophages (left histogram) were pre-incubated for 1 h in the absence or presence LiCl. Cells were then stimulated for 25 minutes with IFN-γ (5 U/ml) or left unstimulated, stained and analyzed for pSTAT1-Y701 in CD11b^+^ gated cells as in (A). CD11b^+^ cells (right histogram) were isolated from dLNs and spleens of MOG_35–55_-immunized mice, restimulated for 24 h with MOG_35–55_ (10 µg/ml) in the absence or presence of LiCl and evaluated for pSTAT1-Y701. (D) Naïve splenocytes from *Ifngr1^−/−^* mice were pre-incubated without or with LiCl, and stimulated for 25 minutes with IFN-γ (5 U/ml) and/or αCD3 (1.25 µg/ml), as indicated, and evaluated for pSTAT1-Y701. Histograms are gated on CD4^+^ T cells.

The IFNGR-STAT1 signaling axis is essential for the differentiation and expansion of Th1 cells in a feed-forward process that involves *Ifng* expression [4], [7], [30]. We therefore investigated if GSK3 facilitated the IFN-γ signaling pathway in T cells by promoting STAT1 activation. Mononuclear cells from spleens of naïve C57BL/6 mice were stimulated with IFN-γ and/or anti-CD3 in the absence or presence of lithium or TDZD-8, a structurally different GSK3 inhibitor, and the extent of pSTAT1-Y701 (pSTAT1) was evaluated in different cell populations. As expected, IFN-γ efficiently induced pSTAT1 in CD4^+^ T cells (Fig. 2A). Anti-CD3 marginally activated STAT1, but costimulation of CD4^+^ T cells with anti-CD3 and IFN-γ resulted in synergistic hyperphosphorylation. Although this synergism has not been previously reported, the result is inferable because T cell receptor (TCR) engagement leads to translocation of IFNGR to the TCR complex within the immunological synapse [31]. Treatment with lithium or TDZD-8, significantly attenuated pSTAT1 induced by stimulation with IFN-γ in presence or absence of costimulation of the TCR (Fig. 2A). Similarly, stimulation with IFN-β also promoted STAT1 activation, and co-stimulation of CD4^+^ T cells with IFN-β and anti-CD3 resulted in the activation of STAT1 that was additive to that induced by IFN-β or anti-CD3 alone (Fig. 2B). Lithium also inhibited STAT1 activation induced by IFN-β with or without anti-CD3 costimulation (Fig. 2B). In agreement with a previous study [19], GSK3 inhibition has no effect on IFN-γ induced activation of STAT1 in macrophages from the peritoneum or spleen (Fig. 2C). However, lithium did inhibit STAT1 activation in B-cells and CD8^+^ T cells (Figs. S2 A, B). As expected, the IFN-γ induced pSTAT1 was absent if IFNGR signaling was genetically ablated (*Ifngr1^−/−^*) (Fig. 2D). However, anti-CD3 stimulation was able to induce STAT1 activation in *Ifngr1^−/−^* mice (Fig, 2 D). Notably, inhibition of GSK3 had no effect on STAT1 activation induced by anti-CD3 alone (Fig. 2A, B, and D). Overall from these data we infer that efficient activation of STAT1 in CD4^+^ T cells by IFN-γ and IFN-β requires active GSK3.

### Lithium Inhibits Th1 Expansion by Tuning IFN-γ-induced STAT1-Y701 Phosphorylation

**Figure 3:**
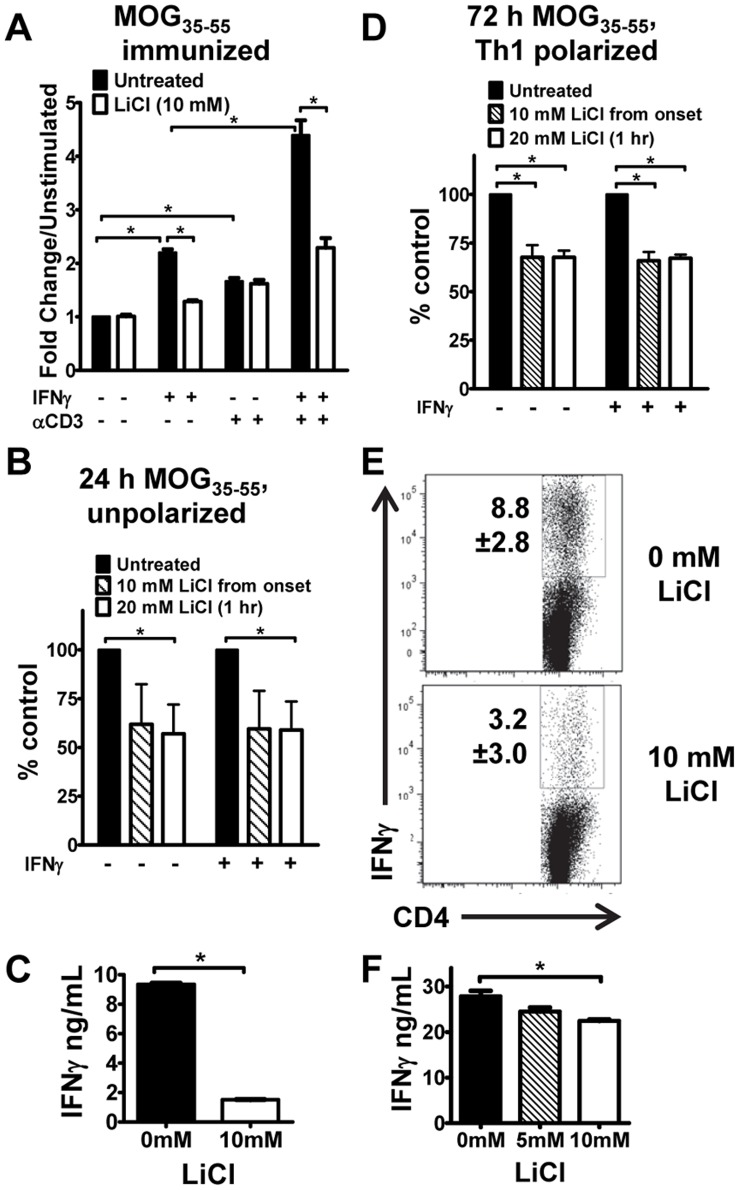
Lithium attenuates STAT1-Y701 phosphorylation in encephalitogenic CD4^+^ T cells and reduces IFN-γ production. (A and B) Cells from dLNs and spleen of MOG_35–55_ immunized mice (10–21 d post immunization) were either (A) pre-incubated without or with LiCl, and left unstimulated or stimulated for 25 with IFN-γ (5 U/ml) and/or anti-CD3 (1.25 µg/ml) as indicated; or (B) restimulated for 24 h with MOG_35–55_ (10 µg/ml) in the absence or presence of LiCl, from onset, or acutely treated for 1 h, as indicated. A subset of cells was stimulated after 24 h with IFN-γ (5 U/ml) for 25 minutes. Cells were gated on CD4^+^ T cells and pSTAT1-Y701 was analyzed as in Fig. 2 and normalized to unstimulated cells from naïve mice. Results are expressed as percent of control, which represent stimulated untreated samples (100%), *n* = 3. (C) IFN-γ production by 24 h MOG_35–55_ restimulated cells (from B). Representative sample shown (*n* = 3). (D) CD4^+^ T cells from spleens and dLNs of MOG_35–55_ immunized mice were polarized under Th1 conditions in the presence or absence of LiCl, from onset or acutely treated for 1 h on day 3. Where indicated cells were stimulated with IFN-γ on day 3. Cells were gated on in CD4^+^, analyzed for pSTAT1-Y701, and normalized to unstimulated cells from naïve mice. Results are expressed as percent of control, which represent stimulated untreated samples (100%), (*n* = 3) (E), Th1 cells generated by polarization in the absence or presence of LiCl. Dot plots reflect CD4^+^ gated T cells. Representative experiment shown (*n* = 2). (F) IFN-γ production from Th1 cells (day 3 of polarization) stimulated with anti-CD3 and anti-CD28 (1 µg/ml each) for 8 h in the absence or presence of LiCl was assessed by ELISA. Representative results shown (*n* = 2). *p<0.05, as determined by t-test or one-way ANOVA, as appropriate.

From the above data we predicted that lithium might modulate encephalitogenic Th1 cells by altering activation of STAT1. In order to test this hypothesis, cells from spleens and dLNs from MOG_35–55_- immunized mice were stimulated with IFN-γ in the presence or absence of TCR engagement (anti-CD3), or with anti-CD3 alone. Both IFN-γ and anti-CD3, each significantly induced pSTAT1 in encephalitogenic T cells, but the co-stimulation with anti-CD3 and IFN-γ resulted in synergistic hyperphosphorylation (Fig. 3A) as observed in naïve cells (Fig. 2A). Lithium attenuated IFN-γ-induced pSTAT1 in the presence or absence of costimulation with anti-CD3 (Figs. 3 A). We next tested the activation of STAT1 in *in vivo* primed T cells restimulated with antigen (MOG_35–55_) (Figs. 3 B, S3 C). The levels of pSTAT1 in unstimulated cells from MOG_33–55_-immunized mice were very low, and did not differ from unstimulated cells from naïve mice (Figs. S3 A, B). Restimulation with MOG_35–55_ for 24 h induced STAT1 activation (Fig. S3 C). Lithium treatment during the 24 h stimulation lowered p-STAT1 levels with respect to untreated control, but the decrease was not statistically significant (Fig. 3B). Remarkably, acute treatment with LiCl (1 h) was sufficient to significantly decrease pSTAT1 (Fig. 3B). This rapid down modulation of pSTAT1 suggests that the target of GSK3 might be a phosphatase, such as SHP2 [32]. Exogenous IFN-γ was unable to further enhance pSTAT1 indicating that STAT1 was maximally activated by endogenous IFN-γ produced during restimulation (Fig. 3B). Notably lithium also dramatically inhibited the TCR-activation dependent production of IFN-γ (Fig. 3C).

We then evaluated if lithium-dependent inhibition of pSTAT1 would affect the generation/expansion of Th1 cells by MOG_35–55_ restimulation under Th1 polarizing conditions (Figs. 3 D, S3 D). We found that the pSTAT1 was much higher in Th1 polarized T cells compared to unstimulated naïve CD4^+^ T cells and that it was not increased further by acute stimulation with IFN-γ (Fig. S3 D). Both continuous and acute lithium treatment significantly inhibited STAT1 activation in Th1 cells (Fig. 3D). Importantly, lithium inhibited the generation of IFN-γ^+^ Th1 cells by more than 50% (Fig. 3E). Acute treatment with 10 mM of lithium at the end of Th1 polarization also significantly inhibited the production of IFN-γ (Fig. 3F). Treatment with 5 mM of lithium, a dose suboptimal for acute exposure, did not have any significant effect on IFN-γ production. Overall, these results demonstrate that lithium suppresses Th1 expansion by modulating IFN-γ-STAT1 activation pathway.

### Intact IFN-γ Signaling is Required for Lithium’s Therapeutic Effectiveness in EAE

**Figure 4:**
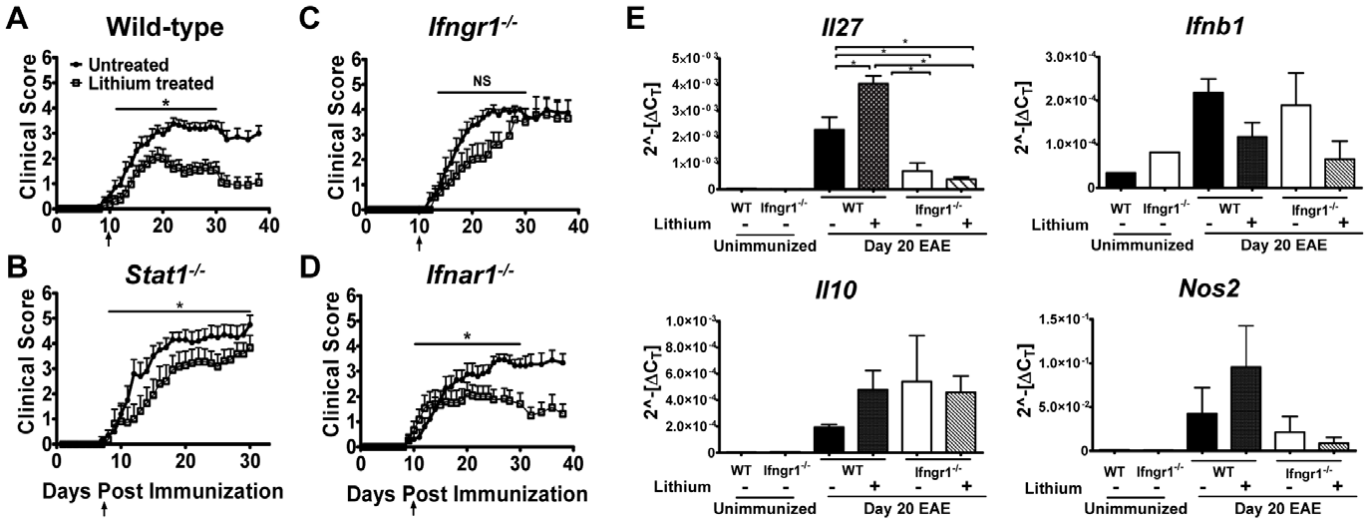
IFN-γ-signaling is required for attenuation of EAE by lithium. EAE was induced in (A) WT (B), *Stat1* ^−/−^, (C) *Ifngr1^−/−^*, and (D) *Ifnar1^−/−^*as described in Material and Methods. Arrows indicate first day of administration of lithium. (Mean ± SEM, *n* = 10–13 mice/group *p<0.05, or NS, not significant, from start of treatment until day 30, as determined by Mann-Whitney test). (E) RNA was isolated from spinal cords of WT and *Ifngr1^−/−^* mice on day 20 post-immunization and evaluated for gene expression by real-time PCR, as detailed in Materials and Methods. *n* = 3 for immunized, *n* = 1 for unimmunized controls. *p<0.05, as determined by one-way ANOVA.

The IFN-γ-STAT1 signaling axis has an important pleiotropic role in autoimmune diseases including MS and its mouse model, EAE [11]. IFN-γ is both pathogenic and protective in autoimmune diseases [4], [11]. These studies suggest that too much or too little IFN-γ signaling can exacerbate autoimmune diseases such as MS; therefore, we posited that lithium balances IFN-γ signals and suppresses EAE by attenuating STAT1 activation. To test this model, we compared the ability of lithium to suppress EAE in wild-type (WT) mice to that in *Stat1* ^−/−^ mice (Figs. 4 A, 4 B). In agreement with our previous study [17], lithium treatment in WT mice beginning day 10 after immunization significantly suppressed clinical disease (Fig. 4A). Lithium treatment caused a more moderate, although significant, attenuation of EAE in *Stat1^−/−^* mice (Fig. 4B), and did delay the onset of disease (Table 2). This suggests the existence of a minor STAT1 independent activity of lithium, a possibility since IFNγR can signal through other STATs [11].

**Table 2:** Analysis of disease parameters for active EAE induced in untreated and lithium-treated WT, *Stat1^−/−^*, *Ifngr1^−/−^* and *Ifnar1^−/−^* mice.

	LiCO_3_diet	Incidence (%)	Onset (d)	Accumulative score
**WT**	–	100% (28/28)	15.2±0.7	54.7±4.1
	+	67% (12/18)	15.1±0.7	29.0±5.0
***Stat1^−/−^***	–	100% (10/10)	12.8±0.8	74.1±5.7
	+	91% (10/11)	17.6±1.8^a^	52.7±9.9
***Ifngr1^−/−^***	–	100% (11/11)	17.1±1.0	43.6±4.3
	+	80% (8/10)	18.9±1.6	28.1±6.9
***Ifnar1^−/−^***	–	100% (13/13)	16.3±1.2	44.1±4.5
	+	75% (9/12)	12.2±1.7^b^	33.2±8.0

Data are presented as mean ± SEM (*n* = 10–28 mice).

a *p*<0.05; Lithium treated *Stat1^−/−^* compared to untreated *Stat1^−/−^*.

b *p*<0.05; Lithium treated *Ifnar1^−/−^* compared to untreated *Ifnar1^−/−^*.

STAT1 is activated by signaling through multiple cytokine receptors, including the IFNGR and the IFNAR [33]. We therefore interrogated if intact IFNGR-STAT1 signaling was necessary for lithium to attenuate EAE. We found that lithium did not significantly suppress EAE in *Ifngr1^−/−^* mice (Fig. 4C). This result indicates that intact IFN-γ signaling is necessary for therapeutic effectiveness of lithium. Unlike in *Ifngr1^−/−^* mice, lithium delayed the onset (Table 2) and effectively attenuated EAE in *Ifnar1^−/−^* mice (Fig. 4D). This result demonstrates that although lithium inhibits STAT1 activation initiated by IFNAR engagement (Fig. 2B), the type I IFN receptor is dispensable for lithium’s therapeutic activity in EAE (Fig. 4D).

### GSK3 Regulates IFN-γ Signaling Dependent Expression of *Il27* in EAE

IL-27 is an IFN-γ response cytokine that has an essential role in limiting EAE by a mechanism that includes, but not limited to the induction of IL-10 in CD4^+^ T cells [34]–[36]. We recently reported that IFN-β promotes the expression of IL-27 in an IFN-γ dependent manner and consequently induces the expression of IL-10 in Th1 cells to limit Th1 EAE [15]. We therefore investigated if lithium treatment induces *Il27* expression in the CNS of mice with EAE. We found that *Il27* expression was upregulated in the spinal cord of WT mice with EAE at the peak of disease and lithium treatment significantly elevated it by approximately 2 fold with respect to untreated mice (Fig. 4E). The induction of *Il27* was much diminished in *Ifngr1^−/−^* mice and was not altered by lithium treatment. IFN-β, a cytokine expressed in the CNS during EAE, can also induce *Il27* expression [37]. We found upregulation of *Ifnb* at peak of disease, but this was not further increased by lithium (Fig. 4E). Therefore our data indicate that GSK3 regulates IFN-γ induced, but not IFN-β-induced, IL-27 expression. *Il10* expression in CNS of WT mice correlated with *Il27* expression (Fig. 4E). Although *Il10* expression in *Ifngr1^−/−^* mice was elevated, its expression levels did not increase with lithium treatment. We also observed that lithium treatment enhanced *Nos2* (inducible nitric oxide synthase) in WT but not in *Ifngr1^−/−^* mice. However the increases in *Il10* and *Nos2* were not statistically significant (Fig. 4E). Overall our data support the tenet that lithium attenuates EAE by an IFN-γ-dependent expression of IL-27.

In conclusion, we discovered a novel mechanism for the therapeutic activity of lithium in EAE that involves the tuning of STAT1 in an IFN-γ-signaling dependent manner. Our study provides new insights in limiting CNS autoimmune disease by regulation of IFN-γ signaling, that is independent of signaling through the receptor for IFN-α/β. These findings will contribute to development of new treatment strategies for neuroinflammatory diseases, as well as offer the opportunity to use GSK3 inhibitors as combined therapy with IFN-β for the treatment of MS to enhance therapeutic effectiveness.

## Supporting Information

Figure S1 **Numbers of CNS-infiltrating cells from Th1 EAE.** (A), Infiltration of Th1 cells in spinal cords and cerebellum of untreated or lithium pretreated passive transfer EAE animals. The CNS cells were isolated from cerebellum and spinal cord at day 14–15 post transfer and characterized by intracellular cytokine staining for IFN-γ expression in CD4-gated cells. (B), Total infiltrating cells in CNS enumerated using a hemocytometer.
(TIF)

Figure S2 **GSK3 mediates IFNγ-induced pSTAT1-Y701 in CD8^+^ T cells, and B220^+^ cells.** (A), B220^+^, or (B*)*, CD8^+^ (CD5^+^CD4^−^) cells were isolated from dLNs and spleens of MOG_35–55_-immunized mice, restimulated for 24 hours with MOG_35–55_ (10 µg/ml) in the absence or presence of LiCl (10 mM). Cells were then evaluated for pSTAT1-Y701 using flow cytometry. Histograms are gated on the indicated populations.
(TIF)

Figure S3 **P-STAT1 in CD4^+^ T cells from MOG_35–55_-immunized mice, and unstimulated cells comparison with cells from naïve mice.** (A) Cells from dLNs and spleen of MOG_35–55_ immunized mice (10–21 d post immunization) were pre-incubated without or with LiCl, and left unstimulated or stimulated for 25 minutes with IFN-γ (5 U/ml) and/or αCD3 (1.25 µg/ml) as indicated. CD4^+^ T cells were stained for pSTAT1-Y701 and analyzed by flow cytometry. Mean fluorescence intensity (MFI) is shown. (*n* = 3). (B) Comparison of levels of pSTAT1-Y701 in unstimulated CD4^+^ T cells from MOG_35–55_-immunized mice with cells from naïve mice. (C) Cells from dLNs and spleen of MOG_35–55_ immunized mice (10–21 d post immunization) were restimulated with MOG_35–55_ (10 µg/ml) for 24 h in the absence or presence of LiCl from onset or acutely treated for 1 h. Additionally, a subset of cells was stimulated after 24 h with IFN-γ (5 U/ml) for 25 min. Histograms are gated on CD4^+^ T cells. (D) CD4^+^ T-cells from spleens and dLNs of MOG_35–55_ immunized mice were polarized for 72 h under Th1 conditions, in the presence or absence of LiCl from onset, or acutely treated for 1 h on day 3. Where indicated, cells were stimulated with IFN-γ on day 3. CD4^+^ T cells were stained for pSTAT1-Y701 analyzed by flow cytometry. Histograms are gated on CD4^+^ T cells.
(TIF)
