# Supplementary figures and images for: Lithium Controls Central Nervous System Autoimmunity through Modulation of IFN-γ Signaling
Source: PLoS One. 2012 Dec 28;7(12):e52658. doi: 10.1371/journal.pone.0052658 (PMC3532311; doi:10.1371/journal.pone.0052658)

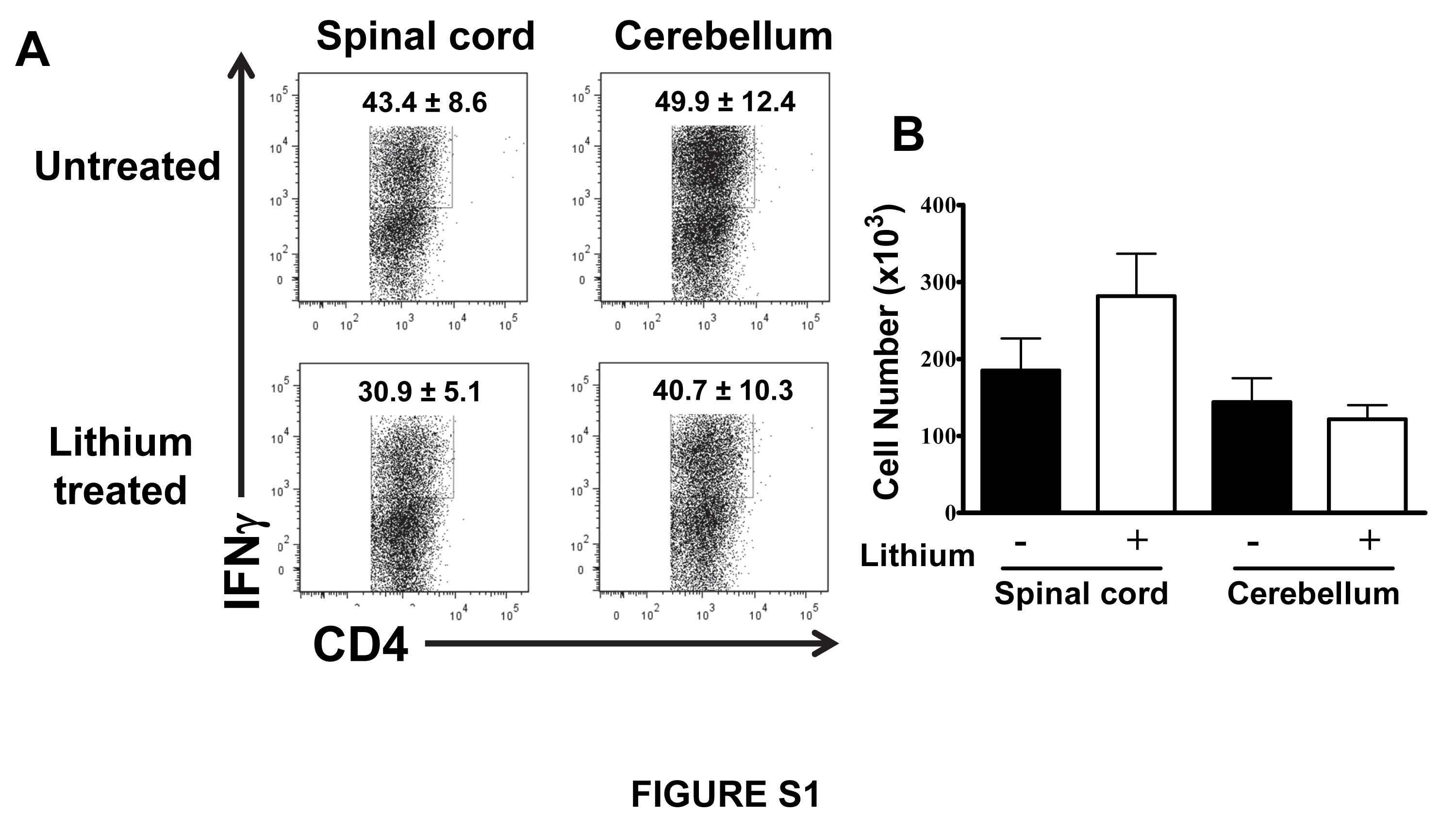

Supplement: Figure S1 — Numbers of CNS-infiltrating cells from Th1 EAE. (A), Infiltration of Th1 cells in spinal cords and cerebellum of untreated or lithium pretreated passive transfer EAE animals. The CNS cells were isolated from cerebellum and spinal cord at day 14–15 post transfer and characterized by intracellular cytokine staining for IFN-γ expression in CD4-gated cells. (B), Total infiltrating cells in CNS enumerated using a hemocytometer. (TIF) [file pone.0052658.s001.tif]

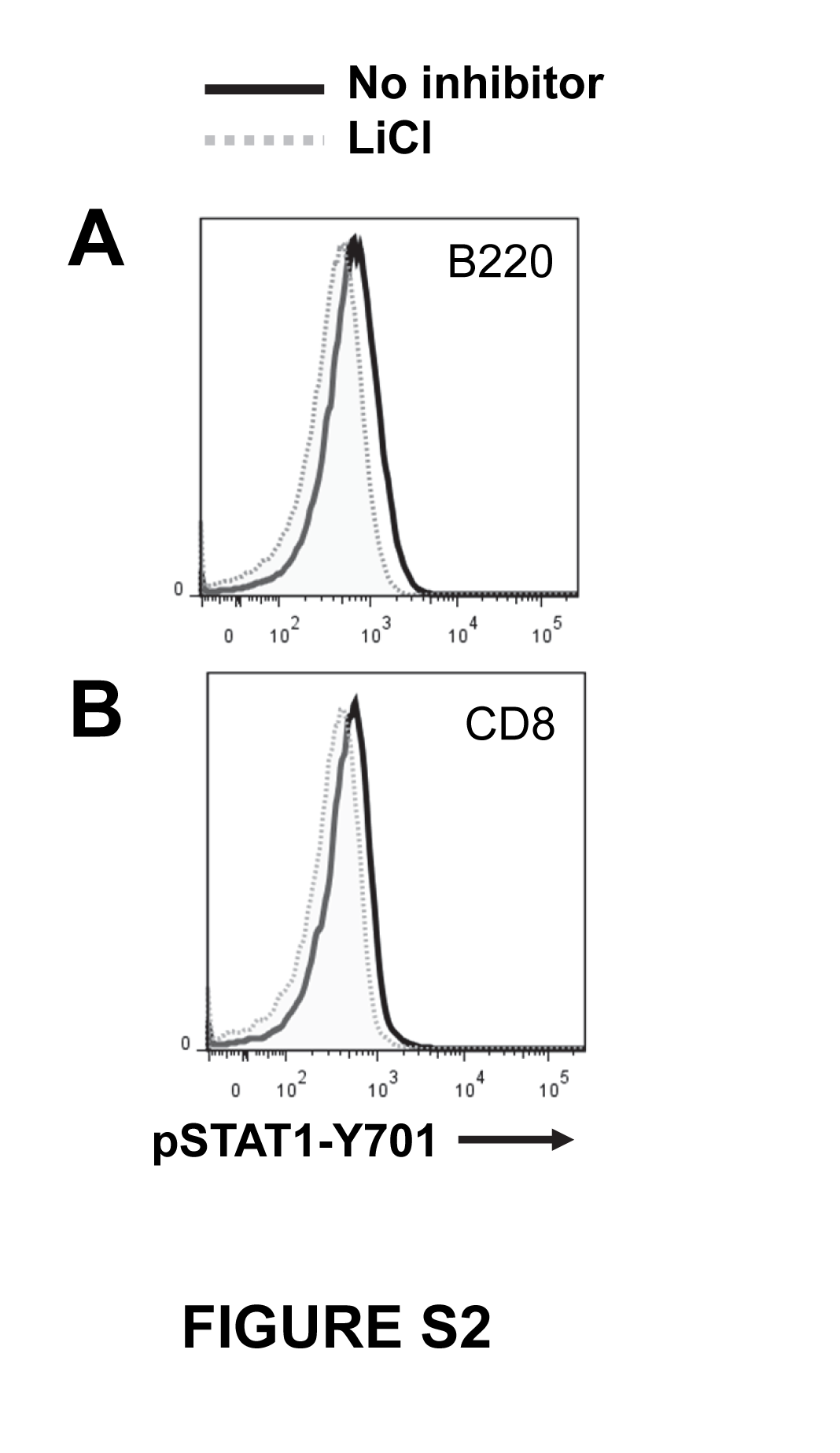

Supplement: Figure S2 — GSK3 mediates IFNγ-induced pSTAT1-Y701 in CD8+ T cells, and B220+ cells. (A), B220+, or (B), CD8+ (CD5+CD4−) cells were isolated from dLNs and spleens of MOG35–55-immunized mice, restimulated for 24 hours with MOG35–55 (10 µg/ml) in the absence or presence of LiCl (10 mM). Cells were then evaluated for pSTAT1-Y701 using flow cytometry. Histograms are gated on the indicated populations. (TIF) [file pone.0052658.s002.tif]

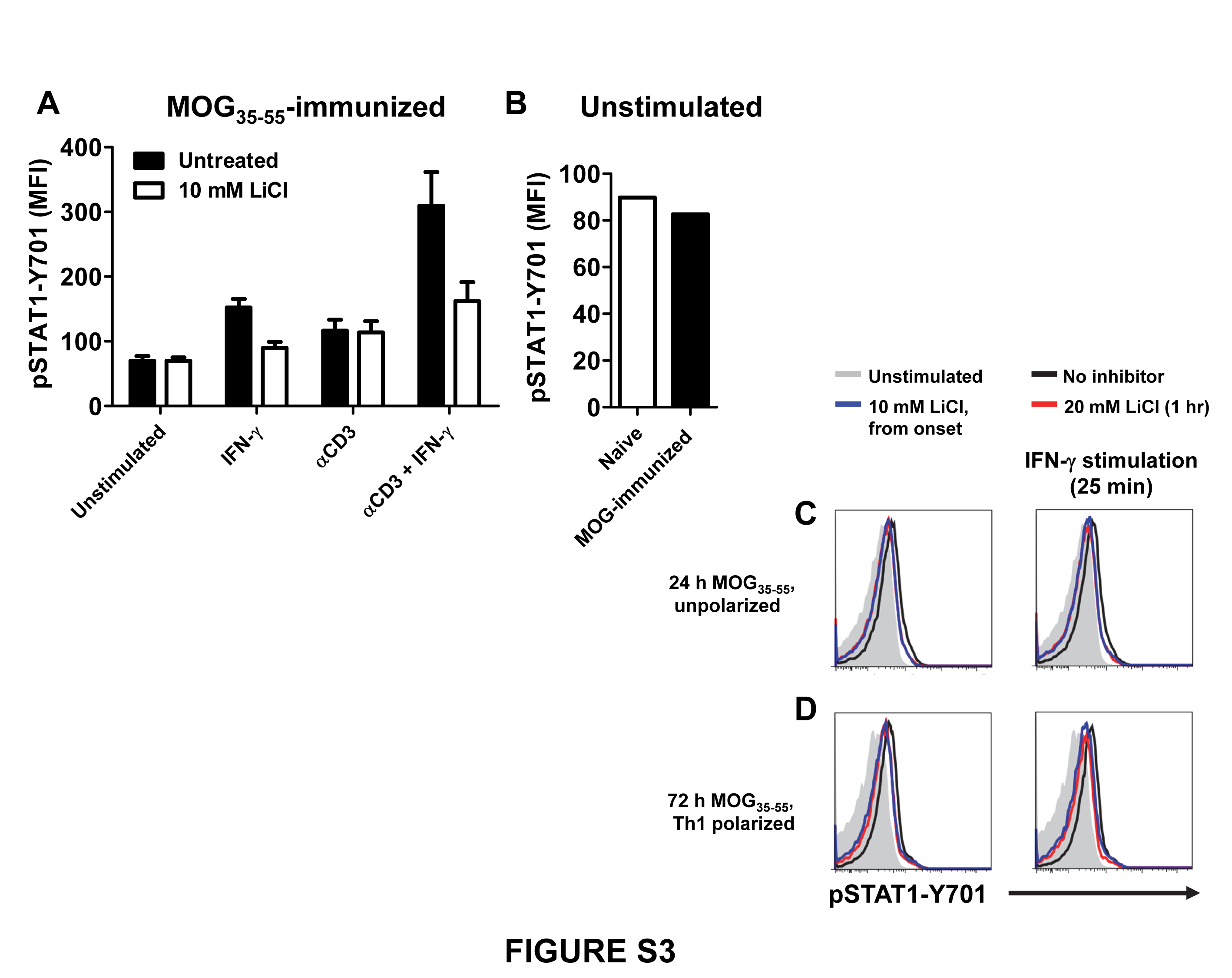

Supplement: Figure S3 — P-STAT1 in CD4+ T cells from MOG35–55-immunized mice, and unstimulated cells comparison with cells from naïve mice. (A) Cells from dLNs and spleen of MOG35–55 immunized mice (10–21 d post immunization) were pre-incubated without or with LiCl, and left unstimulated or stimulated for 25 minutes with IFN-γ (5 U/ml) and/or αCD3 (1.25 µg/ml) as indicated. CD4+ T cells were stained for pSTAT1-Y701 and analyzed by flow cytometry. Mean fluorescence intensity (MFI) is shown. (n = 3). (B) Comparison of levels of pSTAT1-Y701 in unstimulated CD4+ T cells from MOG35–55-immunized mice with cells from naïve mice. (C) Cells from dLNs and spleen of MOG35–55 immunized mice (10–21 d post immunization) were restimulated with MOG35–55 (10 µg/ml) for 24 h in the absence or presence of LiCl from onset or acutely treated for 1 h. Additionally, a subset of cells was stimulated after 24 h with IFN-γ (5 U/ml) for 25 min. Histograms are gated on CD4+ T cells. (D) CD4+ T-cells from spleens and dLNs of MOG35–55 immunized mice were polarized for 72 h under Th1 conditions, in the presence or absence of LiCl from onset, or acutely treated for 1 h on day 3. Where indicated, cells were stimulated with IFN-γ on day 3. CD4+ T cells were stained for pSTAT1-Y701 analyzed by flow cytometry. Histograms are gated on CD4+ T cells. (TIF) [file pone.0052658.s003.tif]
